# Different associations of sufficient and vigorous physical activity with BMI in Northwest China

**DOI:** 10.1038/s41598-018-31227-6

**Published:** 2018-09-03

**Authors:** Feng Liu, Weihua Wang, Jingang Ma, Rina Sa, Guihua Zhuang

**Affiliations:** 1 Department of Epidemiology and Biostatistics, School of Public Health, Xi’an Jiaotong University Health Science Center, Xi’an, China; 2 Shaanxi Center for Disease Control and Prevention, Xi’an, China

## Abstract

Physical activity (PA) plays an important role in maintaining a healthy weight. To develop targeted strategies that encourage physical activity, knowledge of associations between intensity of physical activity (PA) levels and body mass index (BMI) is essential. We aimed to examine the relationship between sufficient and vigorous PA and BMI distribution among adults in northwest China using quantile regression. We conducted a cross-sectional study in Shaanxi Province in 2013, using proportional probability sampling. BMI was calculated using measured height and weight. The Global Physical Activity Questionnaire was used to define and measure sufficient and vigorous PA. Associations of sufficient/vigorous PA and BMI were modelled using quantile regression. Mean BMI was 24.18 ± 3.51 and BMI distribution with age showed an inverse U shape. A total 9045 (88.97%) participants demonstrated sufficient PA and 3119 (30.68%) reported vigorous PA. After adjusting for relevant sociodemographic, dietary, and lifestyle parameters in quantile regression modelling, sufficient PA was positively associated with BMI score distribution from the 1st to 30th quantile, with β from 0.32 (95% confidence interval (CI): 0.07 to 0.63) to 0.85 (95% CI: 0.40 to 1.19). Vigorous PA was negatively associated with BMI score distribution from the 30th to 93th quantiles, with β from −0.18 (95% CI: −0.31 to −0.02) to −0.81 (95% CI: −1.10 to −0.45). Sufficient PA was positively associated with underweight and normal weight whereas vigorous PA was negatively associated with overweight and obesity.

## Introduction

Physical activity (PA) guidelines of the World Health Organization (WHO) recommend moderate- or vigorous-intensity PA for 150 or 75 minutes weekly, respectively. This recommendation aims to promote human health by reducing the potential risks of chronic diseases, such as coronary heart disease, diabetes, and cancer^1–3^. However, the positive effects of preventing weight gain should be examined to decide whether this recommendation is sufficient to overcome overweight and obesity.

There is no evidence that preventing weight gain requires 150 minutes of moderate-intensity PA or lesser amounts of vigorous-intensity PA per week. A cohort study in the United States on women’s health found that with 60 minutes or more of PA per day, weight gain could be prevented only in women with normal weight^4^. By contrast, 30 minutes daily was sufficient to prevent weight gain in young adults in another study^5^. Obesity and malnutrition are not uncommon in China, but there is less evidence proving an association of PA with weight gain prevention^6,7^. Findings from the China Kadoorie Biobank show a 1-standard deviation (SD) (14 metabolic equivalents (MET)-h/d) greater intensity PA is associated with a 0.15-unit lower BMI^8^, whereas results from another study in China showed individuals aged ≥50 years who engaged in greater intensity PA had higher body mass index (BMI)^9^. These studies examined the association of PA as a continuous variable and mean BMI of the study population; however, results from these studies varied among the different included populations. Therefore, the effect of PA on BMI distribution, that is, from underweight to obesity, must be explored. We sought to further investigate the impacts of PA intensity on BMI in China.

To help understand the association of PA and BMI in the Chinese population, especially that of northwest China, we analysed PA data from Shaanxi Province obtained from the Chinese Chronic Diseases and Risk Factors Surveillance Survey (CDRFSS) in 2013^10^. The present study aimed to determine the prevalence of PA and explore the direction and strength of associations between PA and the distribution of BMI.

## Results

### Participant characteristics

Table 1 shows the demographic, dietary, and lifestyle factors of participants in the PA groups. Participants’ age ranged from 18–91 years, with mean age 52.98 years. Of 10,166 participants, 9045 (88.97%) engaged in sufficient PA and 3119 (30.68%) engaged in vigorous PA. Compared with participants who reported sufficient PA, those reporting insufficient PA were more likely to be male (51.03%), older (age 52.98 ± 17.02 years), living in urban areas (61.20%), less educated (≤6 years: 43.98%), and to consume less fresh vegetables/fruit (476.73 ± 415.19 g) but more red meat (44.44 ± 92.5 g) and salt (10.25 ± 10.32 g) per day. Participants with vigorous PA were more likely to be male (62.46%), younger (age 47.59 ± 12.32 year), living in rural areas (60.31%), have ≤6 years of education (46.23%), and to consume more salt (10.4 ± 8.45 g), oils (61.62 ± 32.85 g), alcohol (26.49 ± 53.25 g), and to smoke cigarettes compared with participants who did not report vigorous PA. These differences were statistically significant between the sufficient PA and insufficient PA groups, as well as the vigorous PA and non-vigorous PA groups.

**Table 1:** Sociodemographic characteristics of participants in northwest China with sufficient/insufficient or vigorous/non-vigorous physical activity.

N	Insufficient PA	Sufficient PA	t/χ2	P	Not-vigorous PA	vigorous PA	t/χ2	P
	1121 (11.03)	9045 (88.97)			7047 (69.32)	3119 (30.68)		
gender			16.279	<0.01			530.15	<0.01
Male	572 (51.03)	4040 (44.67)			2664 (37.80)	1948 (62.46)		
female	549 (48.97)	5005 (55.33)			4383 (62.20)	1171 (37.54)		
Residence			34.537	<0.01			316.54	<0.01
rural	435 (38.80)	4350 (48.09)			2904 (41.21)	1881 (60.31)		
urban	686 (61.20)	4695 (51.91)			4143 (58.79)	1238 (39.69)		
age	52.98 ± 17.02	49.11 ± 13.84	−7.31	<0.01	50.4 ± 14.98	47.59 ± 12.32	9.91	<0.01
Education			12.397	0.006			80.72	<0.01
≤6 years	493 (43.98)	3665 (40.52)			2716 (38.54)	1442 (46.23)		
6~9 years	536 (47.81)	4789 (52.95)			3783 (53.68)	1542 (49.44)		
>9 years	89 (7.94)	577 (6.38)			538 (7.63)	128 (4.10)		
vegetable/fruit	476.73 ± 415.19	535.35 ± 425.87	4.358	<0.01	528.15 ± 410.42	530.53 ± 456.51	−0.26	0.79
red meat	44.44 ± 92.50	35.97 ± 74.17	−2.948	0.003	36.9 ± 75.55	36.92 ± 78.44	−0.015	0.99
Salt	10.25 ± 10.32	9.55 ± 7.74	−2.001	0.045	9.27 ± 7.84	10.40 ± 8.45	−5.99	<0.01
Oil	56.07 ± 29.31	57.53 ± 31.79	1.271	0.204	55.45 ± 30.74	61.62 ± 32.85	−7.95	<0.01
alcohol	19.71 ± 50.46	17.91 ± 44.79	−1.144	0.253	14.39 ± 40.99	26.49 ± 53.25	−11.29	<0.01
Smoke			1.768	0.184			326.03	<0.01
Nonhabitual	841 (75.02)	6947 (76.80)			5754 (81.65)	2034 (65.21)		
Habitual	280 (24.98)	2098 (23.20)			1293 (18.35)	1085 (34.79)		
BMI group			6.064	0.194			32.13	<0.01
<18.5	48 (4.28)	271 (3)			225 (3.19)	94 (3.01)		
18.5~24	524 (46.74)	4285 (47.37)			3223 (45.74)	1586 (50.85)		
24~28	384 (34.26)	3197 (35.35)			2513 (35.66)	1068 (34.24)		
≥28	147 (13.11)	1167 (12.90)			978 (13.88)	336 (10.77)		
MET (min/week)	184.89 ± 197.16	7017.79 ± 6798.39	95.265	<0.01	4217.06 ± 4193.22	10889.9 ± 8854.29	−40.14	<0.01

Values are presented as n (%) or mean ± SD.

Abbreviations: PA, physical activity; BMI, body mass index; MET, metabolic equivalent.

### BMI by sex and age groups

**Figure 1:**
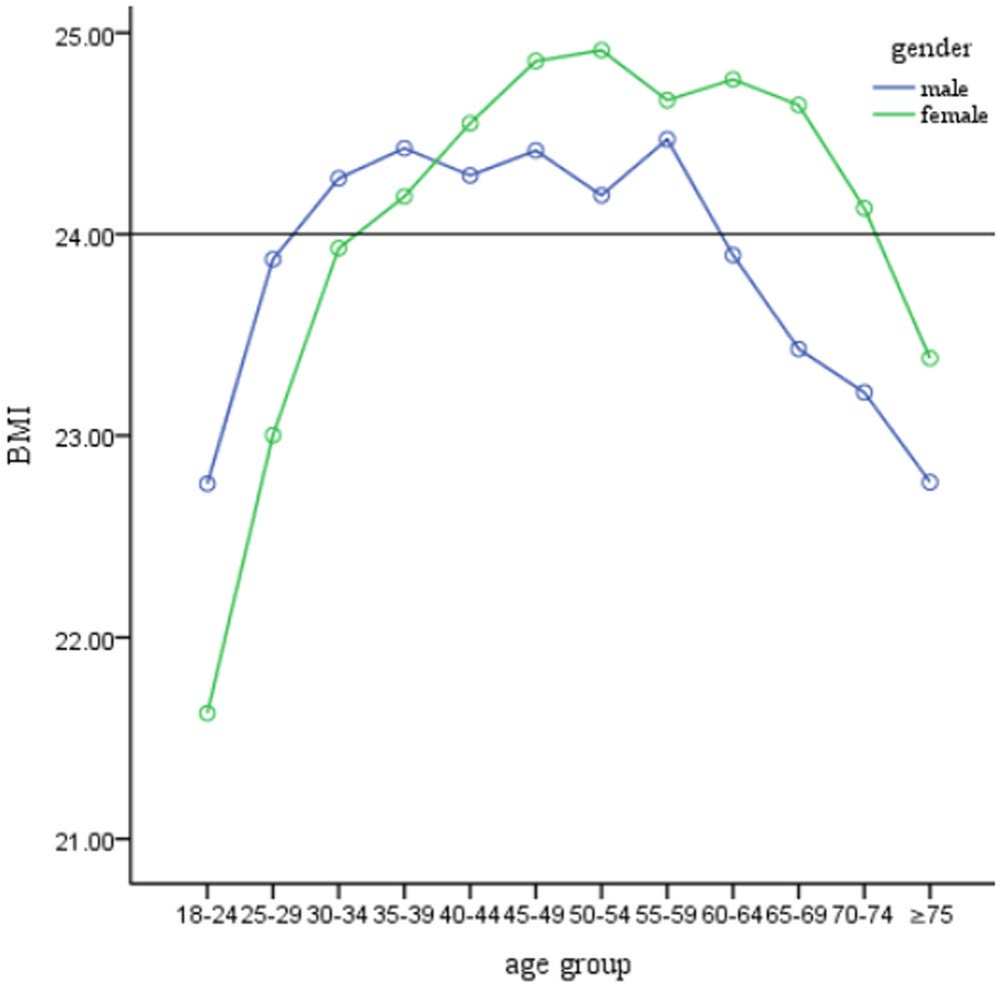
BMI prevalence in northwest China, by age and sex.

Table 2 and Fig. 1 show the mean and median of BMI by sex and age groups. The mean BMI was 24.18 ± 3.51, 24.00 ± 3.36 for males and 24.34 ± 3.63 for females. An inverse U shape was observed with the highest BMI in men aged 55–59 years and women aged 50–54 years.

**Table 2:** BMI prevalence in northwest China by age and sex.

Age group	male			female		
	N	Mean ± SD	Median (25th~75th percentile)	N	Mean ± SD	Median (25th~75th percentile)
18~24	275	22.76 ± 3.44	22.76 (20.28~24.79)	231	21.62 ± 3.27	20.92 (19.31~22.90)
25~29	279	23.88 ± 3.86	23.73 (20.90~25.90)	356	23.00 ± 3.62	22.49 (20.45~24.80)
30~34	264	24.28 ± 3.69	23.96 (22.06~25.90)	348	23.93 ± 3.43	23.37 (21.36~26.17)
35~39	305	24.43 ± 3.38	24.00 (22.02~26.29)	447	24.19 ± 3.71	23.79 (21.52~26.01)
40~44	581	24.29 ± 3.42	23.89 (21.81~26.55)	677	24.55 ± 3.50	24.13 (22.22~26.56)
45~49	570	24.42 ± 3.23	24.27 (22.10~26.36)	766	24.86 ± 3.38	24.65 (22.46~26.94)
50~54	581	24.19 ± 3.23	24.00 (22.05~26.14)	758	24.91 ± 3.56	24.73 (22.35~27.02)
55~59	591	24.47 ± 3.22	24.16 (22.18~26.67)	714	24.67 ± 3.46	24.58 (22.35~26.68)
60~64	487	23.90 ± 3.12	23.78 (21.69~26.09)	516	24.77 ± 3.74	24.54 (22.10~26.90)
65~69	289	23.43 ± 3.26	23.12 (21.03~25.71)	330	24.64 ± 3.69	24.63 (22.04~26.91)
70~74	201	23.21 ± 3.23	22.84 (20.76~25.52)	210	24.13 ± 3.49	23.98 (21.65~26.37)
≥ 75	189	22.77 ± 2.79	22.87 (20.78~24.33)	201	23.38 ± 3.99	23.03 (21.08~25.49)
Total	4612	24.00 ± 3.36	23.80 (21.62~26.08)	5554	24.34 ± 3.63	24.03 (21.73~26.45)

Abbreviations: BMI, body mass index; SD, standard deviation.

### Association between sufficient PA and BMI

QR and OLS results obtained for BMI scores as an outcome variable are reported in Tables 3 and 4. After adjusting for variables from models 2, 3, and 4, the β estimates showed that lower BMI scores were positively associated with sufficient PA whereas central to higher BMI scores had a negative association with vigorous PA.

**Table 3:** Association between sufficient PA and BMI using QR and OLS models for different quantiles of BMI.

		Model1	Model2	Model 3	Model 4
underweight	1st	0.81 (0.33 to 1.25)	**0.66** (**0.51 to 1.45)**	**0.51** (**0.21 to 1.36)**	**0.53** (**0.09 to 1.27)**
	2th	**0.72** (**0.25 to 1.19)**	**0.85** (**0.40 to 1.19)**	**0.68** (**0.28 to 1.04)**	**0.54** (**0.32 to 1.03)**
normal	3th	0.49 (0.24 to 0.99)	**0.70** (**0.50 to 1.23)**	**0.62** (**0.19 to 1.09)**	**0.69** (**0.32 to 1.05)**
	10th	**0.46** (**0.24 to 0.73)**	**0.58** (**0.23 to 0.90)**	**0.57** (**0.25 to 1.01)**	**0.60** (**0.32 to 1.04)**
	20th	**0.35** (**0.11 to 0.56)**	**0.49** (**0.16 to 0.73)**	**0.47** (**0.19 to 0.79)**	**0.55** (**0.27 to 0.87)**
	30th	0.23 (0.02 to 0.45)	0.29 (0.05 to 0.50)	**0.32** (**0.07 to 0.63)**	0.25 (−0.04 to 0.62)
	40th	−0.008 (−0.26 to 0.20)	0.10 (−0.16 to 0.40)	0.21 (−0.12 to 0.49)	0.25 (0.002 to 0.50)
overweight	51th	0.04 (−0.13 to 0.20)	0.10 (−0.14 to 0.30)	0.19 (−0.008 to 0.47)	0.26 (0.01 to 0.45)
	60th	0.01 (−0.13 to 0.26)	0.07 (−0.14 to 0.31)	0.23 (−0.04 to 0.49)	0.22 (−0.09 to 0.49)
	70th	0.14 (−0.05 to 0.33)	0.18 (−0.14 to 0.31)	**0.38** (**0.09 to 0.70)**	**0.40** (**0.13 to 0.60)**
	80th	0.17 (−0.24 to 0.37)	0.06 (−0.28 to 0.37)	0.41 (−0.08 to 0.69)	0.33 (−0.10 to 0.71)
obesity	87th	−0.07 (−0.52 to 0.23)	−0.19 (−0.61 to 0.22)	0.39 (−0.39 to 0.69)	0.28 (−0.25 to 0.56)
	90th	−0.31 (−0.68 to 0.14)	−0.30 (−0.81 to 0.07)	0.11 (−0.55 to 0.54)	−0.09 (−0.64 to 0.53)
	91th	−0.34 (−0.70 to −0.02)	−0.30 (−0.90 to 0.13)	0.05 (−0.67 to 0.41)	−0.15 (−0.65 to 0.37)
	93th	−0.31 (−0.93 to 0.16)	−0.38 (−1.03 to −0.001)	−0.20 (−0.89 to 0.36)	−0.36 (−0.93 to 0.40)
ols		0.10 (−0.11to 0.32)	0.15 (−0.07 to 0.37)	**0.32** (**0.06 to 0.57)**	**0.32** (**0.07 to 0.58)**

Abbreviations: PA, physical activity; BMI, body mass index; CI, confidence interval; OLS, ordinary least squares; QR, quantile regression.

Values are β estimates (95% CI). Coefficients and CI significant at the 5% level are in bold.

**Table 4:** Association between vigorous PA and BMI using QR and OLS models for different quantiles of BMI.

		Model1	Model2	Model 3	Model 4
underweight	1st	0.21 (−0.04 to 0.48)	0.33 (−0.003 to 0.56)	0.23 (−0.25 to 0.42)	**0.23** (**−0.05 to 0.48)**
	2th	0.13 (−0.04 to 0.28)	0.30 (0.02 to 0.56)	0.21 (−0.14 to 0.53)	0.21 (−0.03 to 0.44)
normal	3th	0.05 (−0.18 to 0.30)	**0.34** (**0.05 to 0.53)**	0.19 (−0.04 to 0.55)	**0.34** (**0.004 to 0.49)**
	10th	0.12 (−0.07 to 0.26)	0.18 (0.01 to 0.38)	0.22 (−0.04 to 0.44)	**0.27** (**0.05 to 0.43)**
	20th	−0.02 (−0.16 to 0.11)	0.12 (−0.04 to 0.27)	0.08 (−0.05 to 0.26)	0.17 (−0.02 to 0.33)
	30th	**−0.18** (**−0.31 to −0.02)**	−0.002 (−0.15 to 0.12)	−0.02 (−0.23 to 0.13)	−0.03 (−0.18 to 0.14)
	40th	**−0.35** (**−0.53 to −0.20)**	**−0.18** (**−0.33 to −0.02)**	−0.14 (−0.31 to 0.02)	−0.15 (−0.31 to −0.02)
overweight	51th	**−0.35** (**−0.54 to −0.19)**	**−0.20** (**−0.35 to −0.04)**	−0.17 (−0.36 to 0.01)	−0.12 (−0.33 to 0.02)
	60th	**−0.50** (**−0.67 to −0.36)**	**−0.25** (**−0.41 to −0.08)**	**−0.29** (**−0.44 to −0.11)**	**−0.27** (**−0.45 to −0.10)**
	70th	**−0.49** (**−0.64 to −0.33)**	**−0.30** (**−0.46 to −0.12)**	**−0.36** (**−0.57 to −0.15)**	**−0.34** (**−0.53 to −0.12)**
	80th	**−0.52** (**−0.67 to −0.29)**	**−0.27** (**−0.47 to −0.10)**	**−0.27** (**−0.47 to −0.007)**	−0.23 (−0.46 to −0.06)
obesity	87th	**−0.50** (**−0.72 to −0.29)**	**−0.41** (**−0.60 to −0.15)**	−0.27 (−0.60 to 0.007)	**−0.36** (**−0.59 to −0.09)**
	90th	**−0.66** (**−0.87 to −0.39)**	**−0.54** (**−0.74 to −0.26)**	**−0.45** (**−0.76 to −0.16)**	**−0.52** (**−0.83 to −0.26)**
	91th	**−0.66** (**−0.91 to −0.46)**	**−0.54** (**−0.79 to −0.28)**	**−0.55** (**−0.84 to −0.16)**	**−0.57** (**−0.90 to −0.24)**
	93th	**−0.81** (**−1.10 to −0.45)**	**−0.70** (**−0.92 to −0.37)**	**−0.63** (**−0.94 to −0.31)**	**−0.62** (**−0.96 to −0.26)**
OLS		**−0.35** (**−0.50 to −0.20)**	−0.15 (−0.31 to 0.008)	−0.16 (−0.34 to 0.02)	−0.14 (−0.32 to −0.04)

Abbreviations: PA, physical activity; BMI, body mass index; CI, confidence interval; OLS, ordinary least squares; QR, quantile regression.

Values are β estimates (95% CI). Coefficients and CI significant at the 5% level are in bold.

As shown in Table 3, the association between lower BMI scores and sufficient PA was statistically significant and robust. The effect of sufficient PA decreased from lower (2nd quantile) to central (20th and 30th quantiles) values of BMI distribution. In practical terms, the QR results suggested that sufficient PA had a greater effect on participants with lower BMI scores, that is, greater than or equal to the 20th quantile (β = 0.35). This trend was also found in other models (models 2, 3, and 4).

The OLS results in models 3 and 4 showed that, controlling for dietary and lifestyle factors, mean BMI in the sufficient PA group was 0.32, which was higher than that of the insufficient PA group, and sufficient PA was positively associated with lower BMI score (underweight and normal weight).

### Association between vigorous PA and BMI

In Table 4, an inverse association between vigorous PA and central and higher BMI was clearly observed. In model 1, the inverse effect of vigorous PA increased from the central (30th quantile) to the higher end (93th quantile) of the BMI distribution.

Given that demographic, dietary, and lifestyle factors may confound the association of BMI with vigorous PA, we repeated the analysis by controlling these confounders in models 2, 3, and 4. The negative relationship in the central and higher quantiles of BMI persisted, but the magnitude of the effects decreased and remained steady after controlling for dietary and lifestyle factors (model 1: β from −0.18 to −0.81 vs. model 4: β from −0.27 to −0.62).We found a considerable reduction (13%–83%) in the magnitude of association between vigorous PA and BMI score at central and higher quantiles (Table 4, model 4 vs. model 1). The results indicated that participation in vigorous PA is negatively related to BMI score and the magnitude is greater for higher BMI scores.

## Discussion

By assessing PA as two categorical indicators (sufficient/insufficient and vigorous/non-vigorous) using quantiles of BMI in the population of northwest China, we found that sufficient PA was positively associated with underweight and normal weight whereas vigorous PA was negatively associated with overweight and obesity. After adjusting for relevant sociodemographic, dietary, and lifestyle factors in QR modelling, results were generalized to the entire distribution of BMI. The positive relationship between sufficient PA and underweight with lower BMI score and negative relationship between vigorous PA and obesity with higher BMI score could still be observed. In addition, significant sociodemographic variations of PA in northwest China were found in our study.

The association between PA and weight found in this study was inconsistent with results reported in previous studies. Findings from a prospective cohort study including 34,079 women in the United States showed that PA was associated with less weight gain among women with BMI < 25^4^. The results of that cohort study supported the findings of the present study that BMI < 25 would increase with sufficient PA over time. In addition, 60 minutes of moderate-intensity PA was found to maintain normal weight in that cohort study, but greater intensity PA was warranted for individuals who were overweight or obese to lose weight, as reported in the present study. Another prospective investigation in the United States showed that PA was independently negatively associated (−1.76 lb across quantiles, P < 0.001) with weight^11^. In that study, instead of classifying PA and BMI into a categorical indicator as in the present study, PA was assessed by means of average energy expenditure per week, and weight changes were evaluated as absolute changes. In addition, details of the effects of PA intensity levels on the distribution of BMI could not be deduced, as in our study.

The present findings showing a negative association between vigorous PA and BMI were similar to those of a prior study from the China Kadoorie Biobank, which found that greater intensity PA was associated with lower BMI^8^. A 1-SD (14 MET-h/d) greater intensity PA was associated with a 0.15-unit lower BMI in that cross-sectional analysis^8^. A dose–response relationship between PA and BMI was found in that study using multiple linear regression; however, whether the inverse association was uniform throughout the BMI distribution, constrained by the regression model applied, could not be investigated. Another study in China found that women or individuals aged >50 years had higher PA level with increased BMI, but no significant association of PA and BMI was found in other subgroups^9^. Participants in that study came from three urban areas of Hangzhou, which limited its comparison with other studies. Furthermore, moderate and vigorous PA showed decreased odds of overweight among Chinese youth in both cross-sectional and longitudinal analyses, showing a similar trend to our study results for vigorous PA and BMI^12^. Some studies have focused on occupational PA, domestic PA, or leisure-time (LT) PA^13,14^. Increased occupational PA resulted in lower weight in both men and women and increased domestic PA resulted in lower body weight among men, in a longitudinal observational study among Chinese adults^13^. Despite increased participation in LTPA, increases in overweight or obesity among Chinese adults were observed from 2000 to 2014 in four national surveys (2000, 2005, 2010, and 2014)^14^. These cohort studies on subgroups of the population or subtypes of PA showed an association of PA with BMI but failed to assess the association of PA with the distribution of BMI, as in our study.

The present cross-sectional study indicated an association between PA and BMI among residents of northwest China; however, the causality is unclear. Some studies have reported that the association between PA and BMI is influenced by socioeconomic factors, dietary patterns, and lifestyle^11,15–17^. However, before and after adjusting for these relevant factors, variation of the β estimates between PA and BMI was stable at either the lower quantiles or higher quantiles of BMI in our study. For the first time, we observed different associations of PA with the distribution of BMI in the Chinese population. In a randomized controlled trial for weight loss, participants lost weight but regained weight over 30 months^18^. In another randomized trial with extensive counselling on diet and exercise, participants had lost weight at 6 months but the weight loss could not be sustained at 24 months^19^. In both trials, the effect of PA on weight loss was seen for a short time, followed by weight gain in overweight and obese participants but not in underweight or normal weight ones. This might be explained by participants with sufficient PA had increased appetite and consumed more food than those with BMI at lower quantiles. At the same time, the increased energy expenditure owing to sufficient PA might not be enough to counter the energy intake required to undertake this level of PA. In this way, people who are underweight or normal weight might gain weight. However, vigorous PA could increase energy expenditure more than sufficient PA, resulting in weight loss.

Our study suggested two important points for weight control. First, once underweight, a person might gain weight with sufficient PA. If people who are underweight engage in more sufficient-level PA, it is likely that they will have better mental and physical health^20–22^. Second, sustained vigorous PA (at least 10 minutes duration) might prevent weight gain. Measures and policies should be encouraged and implemented for those who are overweight and obese and less likely to engage in vigorous PA.

The main strength of the present study was the use of QR rather than more commonly used OLS estimates. From the minimum to maximum response, this regression for modelling BMI scores offered a more comprehensive picture of the relationship between variables. Given that multiple quantiles could be modelled, it was possible to achieve a more complete and robust understanding of how BMI score distributions are affected by PA. Other strengths of our study include large-scale recruitment of individuals living in northwest China. PA in our study was investigated with the GPAQ, which is a suitable and accepted instrument for monitoring PA in population health surveillance systems. The data were cleaned and analysed according to the GPAQ analysis guide recommended by the WHO^23^. We also adjusted for many variables that could potentially confound the relationship between PA and BMI. These measurements improved the validity and reliability of the study.

The study also had some limitations. First, the relationship between PA and BMI was estimated according to a cross-sectional design, making it difficult to establish a causal association. Second, health surveillance was carried out in one province of northwest China and selection bias might be present, which might restrict applicability of the results to broader populations. Third, although red meat and fresh fruit and vegetables were measured using a FFQ, no typical food items were included in the questionnaire, which prevented us from calculating the energy intake and overall fibre intake. Energy intake could not be controlled in the final model when we investigated the association between PA and BMI. However, our findings have important implications for further prospective studies on this approach. Our application of QR models offers a novel and relevant approach to investigating the association of PA and BMI.

In this study, we found that sufficient PA was positively associated with underweight and normal weight whereas vigorous PA was negatively associated with overweight and obesity. Different intensities of PA might be recommended by healthcare workers, according to people’s BMI score.

## Methods

During June to August, 2013, a cross-sectional survey was conducted in Shaanxi Province, located in northwest China. Multistage (four-stage) cluster sampling was used to select a provincially representative sample of the adult population (age ≥18 years). Seventeen of 108 counties/districts in Shaanxi Province were selected, stratified by geographic distribution (north, central and south) and by area (urban vs. rural). Three townships (in rural areas) or two streets (in urban areas) were selected from each selected county/district using a proportional probability sampling (PPS) method. Three villages/neighbourhoods from each sampled township/street were also selected using PPS. One hundred participants were then selected by random sampling in every selected village/neighbourhood. A total of 10,320 participants were sampled^10^. The term urban in this study refers to a district in a city and rural means a county according to criteria of the China National Statistics Bureau.

A detailed description of the CDRFSS is given elsewhere^10^. A close-ended questionnaire was administered by public health professionals, trained according to a standard protocol and required to pass a performance exam to be qualified for data collection. Information on demographics (sex, age, education, occupation), diet (meat, fresh vegetables and fruit, oils, salt), lifestyle (smoking, alcohol use, PA) related to BMI was collected.

Blood samples were collected from all participants after an overnight fast of at least 10 hours, and physical measurements were taken including height, weight, waist circumference, blood pressure. Of 10,320 participants, 10,166 with complete data were finally included in this analysis (98.5%). The survey received ethical approval from the Ethical Review Committee of the Chinese Center for Disease Control and Prevention and was carried out according to the Declaration of Helsinki. All participants provided written informed consent.

### Study variables

Three domains of PA were collected, including work, travel and recreational time, based on the Global Physical Activity Questionnaire (GPAQ)^24^, which has been tested for reliability and validity (Kappa 0.67 to 0.73, Spearman’s rho 0.67 to 0.81)^25^. Data cleaning and analysis of PA was conducted, adhering to the GPAQ analysis guide^23^. Participants were asked if they engaged in vigorous-intensity PA during a typical week for at least 10 minutes at a time. If they responded ‘yes’, the number of days per week and the average total time per day spent doing these activities were then queried. Similar questions were asked to determine participants’ participation in moderate-intensity PA. PA recommendations of the WHO for health were considered to be satisfied if participants reported engaging in at least 150 minutes of moderate-intensity PA or 75 minutes of vigorous-intensity PA per week, or an equivalent combination of moderate- and vigorous-intensity PA totalling at least 600 MET-minutes per week. We classified PA level in this study as sufficient versus insufficient PA or vigorous versus non-vigorous PA, according to the GPAQ analysis guide as follows: PA was considered sufficient if participants reported PA levels equal to or above the WHO recommendation and insufficient if participants reported less than the WHO recommendation. Vigorous PA was defined as participants engaging in vigorous-intensity PA that causes large increases in breathing or heart rate, such as carrying or lifting heavy loads or digging or construction work, for at least 10 minutes continuously; non-vigorous PA was if participants did not engage in such PA.

Anthropometry data on participants were collected by trained public health professionals. Participants’ height was measured in metres using a measuring device with 1-mm precision (Model TZG; Wuxi Weigher Factory Co., Ltd.) and weight was measured using an electronic scale with 100-g precision (HD-390; TANITA Corporation). All scales were calibrated before measurement. BMI was calculated as weight divided by the square of height (kg/m^2^) and categorized as underweight (<18.5), normal weight (18.5–23.9), overweight (24–27.9), and obesity (≥28)^26^.

Sociodemographic characteristics included participants’ age (years) at the time of the survey, and years of education (≤6 years, 6–9 years, ≥9 years). A food frequency questionnaire was used to collect habitual dietary intake of participants by asking the frequency of consumption and portion size of typical food items over the previous 12 months. Habitual smoking was defined as smoking every day. The daily quantity of pure alcohol consumed (g) was calculated by multiplying the percentage ethanol content by the quantity or bottles of alcoholic beverages consumed in a day.

### Statistical analysis

We first described characteristics of the two PA groups (sufficient/insufficient, vigorous/non-vigorous) using mean ± SD or n (%) and then compared them using a *t*-test or χ^2^ statistical test. BMI classified by sex and age group was described using mean ± SD and median with interquartile range (IQR).

To understand the association between PA and different BMI scores, we used quantile regression (QR) to estimate and conduct inference from lowest to highest BMI scores. QR offers a mechanism for estimating the conditional quantile of the distribution of BMI scores. Unlike traditional linear regression based on ordinary least squares (OLS) explaining the mean of the BMI, QR can explain the determinants of BMI at any point on its distribution. Therefore, the relationships between sufficient PA and BMI or between vigorous PA and BMI were examined using the multivariate QR model. Fifteen quantiles of BMI scores were selected (1st, 2nd, 3rd, 10th, 20th, 30th, 40th, 51th, 60th, 70th, 80th, 87th, 90th, 91th, and 93th), from lowest to highest BMI, in which the 3rd, 51th and 87th were the cut-offs for underweight, normal, overweight, and obesity. The association between sufficient PA and BMI or between vigorous PA and BMI was explored at underweight (1st, 2nd, 3rd), normal (10th, 20th, 30th, 40th, 51th), overweight (60th, 70th, 80th, 87th), and obesity (90th, 91th and 93th) quantiles.

Regression coefficients for each quantile and 95% confidence intervals (CIs) were estimated for sufficient PA and vigorous PA using insufficient PA and non-vigorous PA as reference, respectively. Four models containing covariates were established step by step for each quantile to control for confounders. Model 1 explored the crude association between PA and BMI. Model 2 adjusted for age, sex, education, and residence. Model 3 further adjusted for dietary intake including consumption of red meat, fresh vegetables and fruit, oils, and salt. Model 4 adjusted for the variables in model 3 plus lifestyle factors including alcohol intake and smoking. R 3.3.1.1 was used for all statistical analyses.
